# Association of matrix metalloproteinase-12 polymorphisms with chronic obstructive pulmonary disease risk: A protocol for systematic review and meta analysis

**DOI:** 10.1097/MD.0000000000021543

**Published:** 2020-07-31

**Authors:** Hongjing Yang, Chuantao Zhang, Jianying Wu, Wei Xiao, Xiaohong Xie, Zhu Zeng, Keling Chen, Wujun Wang, Xing An, Wenjun Tang, Qingsong Huang

**Affiliations:** a Department of Respiratory Medicine; b Department of Gastroenterology, Hospital of Chengdu University of Traditional Chinese Medicine, Chengdu, China.

**Keywords:** chronic obstructive pulmonary disease, meta-analysis, matrix metalloproteinase 12, polymorphism

## Abstract

**Background:**

Chronic obstructive pulmonary disease (COPD) is a multifactorial disease with gene-environment interaction leading to airflow limitation through the respiratory tract. Reports on the association of matrix metalloproteinase 12 (MMP-12) polymorphisms with COPD have been controversial. A new systematic evaluation which could examine whether MMP-12 mutations are associated with the susceptibility to COPD is needed.

**Methods:**

We will search PubMed, EMBASE, Web of Science, China National Knowledge Infrastructure and Google Scholar to obtain eligible case-control studies for meta-analysis. The time is limited from the construction of the library to July 2020. Two investigators systematically will extract relevant data within those included studies.

The odds ratio and 95% confidence intervals will be used to assess the genetic association between the allelic, dominant and recessive models of MMP-12 gene polymorphisms and COPD risk. Stata 12.0 software and Revman 5.3 will be adopted for statistical analysis. This protocol reported under the Preferred Reporting ltems for Systematic Reviews and Meta-Analyses Protocols statement.

**Results:**

This study will provide a better understanding of the association between MMP-12 polymorphisms and COPD risk.

**Conclusion:**

Publishing this protocol will minimise the possibility of bias due to post hoc changes to the analysis protocol.

## 1 Introduction

Chronic obstructive pulmonary disease (COPD) is a common disease with persistent respiratory symptoms and airflow limitation. COPD is characterized by symptoms including progressive dyspnea, cough, and expectoration and can lead to adverse outcomes, such as osteoporosis, heart disease, anxiety, depression, and lung cancer.^[1]^ Although COPD is preventable and treatable, it has 1 of the highest morbidity and mortality rates in the world. About 384 million people worldwide suffer from COPD,^[2]^ of which China accounts for 100 million.^[3]^ In 2015, 3.2 million deaths due to COPD were reported, that is, an 11.6% increase in mortality from 1990.^[4,5]^ Hence, COPD exerts enormous economic burden on the society and is likely to remain as a major health care issue in the coming decades.^[6,7]^

COPD is a complex multifactorial disease and its development is thought to be largely under genetic control. A considerable number of genes and polymorphisms have been identified as possible candidates for COPD risk.^[8–12]^ The gene encoding matrix metalloproteinase 12 (MMP-12) is 1 such candidate. Overexpression of MMP-12 can lead to pathological extracellular matrix protein degradation and excessive airway remodeling, and it may be an important factor in the development of COPD. Extensive evidence from genetic studies, animal models and human diseases suggests that MMP-12 derived from alveolar macrophages plays an indispensable role in lung destruction of COPD.^[13–17]^ Two functional single nucleotide polymorphisms (SNPs) were described in the MMP-12 gene: the (–82) A/G (rs2276109) and Asn357Ser (A/G) (rs652438) polymorphisms are located in the promoter region and haemopexin domain of the enzyme, respectively, and may influence MMP-12 gene expression and activity.^[18,19]^ A case-control genetic association study identified the 2 functional SNPs were associated with severe and very severe COPD (GOLD stages III and IV).^[20,21]^ However, other studies have found that MMP-12 polymorphism are not associated with COPD risk.^[13,22–24]^ Therefore, the association between MMP12 polymorphism and COPD remains unclear. Although a similar systematic review based on COPD was published in 2014,^[25]^ new studies have occurred since. In light of the small sample sizes and limited statistical capacity of individual studies, we will conduct a systematic review and meta-analysis of the existing literature to provide a more comprehensive and precise estimate of the association between MMP-12 polymorphism and COPD risk.

## 2 Methods/design

### 2.1 Study registration

The protocol has been registered in the Open Science Framework (OSF) (registration number: DOI 10.17605/OSF.IO/KNGJC). This systematic review and meta-analysis will be reported in accordance with the preferred reporting items for systematic reviews and meta-analysis protocols 2015.^[26]^ Ethical approval is not required for the study.

### 2.2 Inclusion criteria

#### 2.2.1 Types of studies

Case-control study related to the susceptibility of MMP-12 polymorphisms to the COPD will be incorporated in our review. And these studies provide the available genotype frequencies for the case and control groups to estimate the odds ratio (OR) and its 95% confidence interval. No restriction will be put on the language, publication date or status of the study.

#### 2.2.2 Types of participants

Participants affected by COPD will be included in the meta-analysis. Control subjects should be defined as healthy subjects without history of COPD. No restrictions will be placed on age, gender, or country.

#### 2.2.3 Outcome

COPD risk comparsions.

### 2.3 Exclusion criteria

Repeat report, conference abstracts, case reports, review paper, or animal study, or study has insufficient data for genotyping distribution calculation or which MMP-12 demonstrated a departure from Hardy-Weinberg equilibrium (HWE) in controls will be excluded. If duplicated studies reporting overlapping data are identified, the most comprehensive 1 will be included in the meta-analysis.

### 2.4 Search strategy

Publications will be searched through Pubmed, Web of Science, Embase, Google Scholar and China National Knowledge Infrastructure (CNKI) databases up to July, 2020. A combination of Medical Subject Headings (MeSH) alongside free terms will be used to hunt all the potentially eligible publications without any language restriction. The following terms will be utilized (“SNP” or “mutation” or “genetic polymorphism” or “variation” or “polymorphism” or “single nucleotide polymorphism” or “variant”) and (“chronic obstructive pulmonary disease” or “COPD” or “chronic obstructive airway disease” or “chronic obstructive lung disease” or “chronic airflow obstruction”’) and (“matrix metalloproteinase 12” or “MMP12”). We also will examine the cross-references in the retrieved studies for publications that were missed in the above search.

### 2.5 Data collection and analysis

#### 2.5.1 Selection of studies

**Figure 1:**
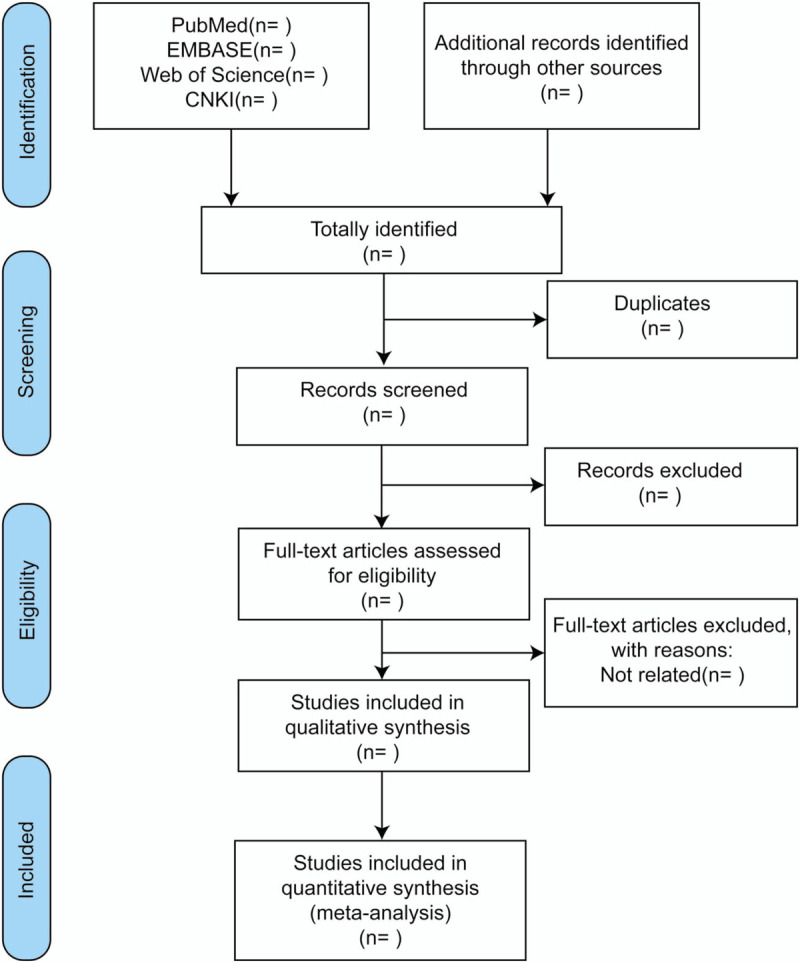
Flow chart of study selection. CNKI = China National Knowledge Infrastructure.

The article screening process will involve reading the title first, followed by the abstract and full text to determine whether the study should be included. Apart of the reviewers in our team will be trained regarding the purpose and process of the review. Two reviewers (Chuantao Zhang and Yang Hongjing) will conduct the selection process independently, with cases of disagreement resolved consulting a third reviewer (Qingsong Huang). A flow chart of study selection is shown in Figure 1.

#### 2.5.2 Data extraction and quality assessment

Data extracted from all qualified articles include: surname of the first author, year of publication, country where the study was performed, ethnicity of enrolled subjects, sample size, the value of HWE, sex composition, mean age, genotyping methods and frequencies of genotypes. All included studies will be evaluated using the Newcastle–Ottawa Scale (NOS).^[27]^ The NOS values arrange from 0 to 9. The studies will be included if the NOS values ≥ 6. Two reviewers (Chuantao Zhang and Yang Hongjing) will conduct the rating independently and a third reviewer (Qingsong Huang) will be consulted for consensus if disagreement occurred. The methodological quality of data will be evaluated strictly in accordance with the STrengthening the REporting of Genetic Association Studies statement.^[28]^

#### 2.5.3 Dealing with missing data

We will try to contact the corresponding authors if the information of potential studies is missing, insufficient, or ambiguous. However, the studies will be excluded if the data cannot be obtained by the above method.

#### 2.5.4 Statistical analysis

Statistical analysis for the meta-analysis will be conducted by Stata version 12.0 (Stata Corporation, College Station, TX) and Revman 5.3 (Cochrane Collaboration). Departure from HWE will be evaluated by using Chi-square test to assess goodness of fit in control subjects of each included study. In order to avoid an inflated type I error rate, we will not perform any assumptions about the genetic model of inherence in advance. The ORs and 95% confidence intervals will be used to evaluate the association between the allelic, dominant and recessive models of MMP-12 and risk of COPD. Then the most plausible genetic model of inherence will be determined according to the relationships between the 3 pairwise comparisons. After that the underlying genetic model is confirmed, the counts of genotypes will be collapsed into 2 categories to obtain the merged results. The significance of the pooled OR will be assessed by the *Z* test, and *p*_Z_ < .05 will be considered as statistically significant.

#### 2.5.5 Assessment of heterogeneity

Heterogeneity will be quantified with the *I*^2^ statistic and *P* value; a *I*^2^ statistic < 50% and a *P* > .1 indicated low heterogeneity among studies, and a fixed model will be applied to estimate the pooled ORs. Otherwise the random model will be used. Subgroup analysis, meta regression analysis, sensitivity analysis will be undertaken to explore potential sources of heterogeneity across studies when statistical heterogeneity is detected.

#### 2.5.6 Subgroup analysis

We will conduct subgroup analyses of the relationships between MMP-12 genetic polymorphisms and the risk of COPD, according to country, ethnicity, and genotyping method, etc.

#### 2.5.7 Sensitivity analysis

Sensitivity analysis will be conducted by removing studies that do not conform to HWE to check the robustness and reliability of pooled outcome results.

#### 2.5.8 Assessment of publication biases

The funnel plots will be utilized to analyze the potential publication bias if there are more than 10 studies. Egger regression test and Begg rank correlation test will be also used to evaluate the publication bias.

#### 2.5.9 Grading the quality of evidence

The quality of evidence for outcome will be assessed by the Grading of Recommendations Assessment, Development, and Evaluation (GRADE) working group approach. High, medium, low, or very low quality represent the 4 levels of evaluation.

## 3 Discussion

COPD is the most common chronic respiratory disease characterized by high morbidity, high disability rate, high mortality and high disease burden. Patients with COPD may need more social and nursing care. The increased social and economic burdens associated with COPD worldwide make their targeting treatment a major public health goal.^[29]^ In general, COPD is a multifactorial disease that can be attributed to an interplay of genetic and environmental factors. Although the specific pathogenesis of COPD is unclear, it is widely accepted that airway tissue remodeling results from disorder of the proteinase-antiproteinase balance and aberrant inflammation in the lung.^[30]^ Among various proteinases, MMP-12 have been shown to play a predominant role in the occurrence and progression of COPD.^[31,32]^ Elevated MMP12 may lead to imbalance of protease-antiprotease and degradation of lung extracellular Matrix, thereby increasing individual's susceptibility to COPD. The genetic polymorphism of MMP-12 gene may induce the abnormal expression of MMP12, and may also be related to the occurrence of COPD.^[13]^ Although the results from a meta-analysis conducted in 2014 suggest that genetic polymorphisms in MMP12 gene may be strongly implicated in the development of COPD,^[25]^ some studies since then have not supported this view.^[23–24]^ the association between MMP12 polymorphism and COPD remains unclear. It is necessary to update previous systematic review about the genetic polymorphism of MMP-12 gene and the risk of COPD MMP-12, which can provide a further reference for the diagnosis and potential therapeutic targets of COPD.

The advantages of this review will be:

(1) this review will include more clinical studies than the previous 1, since only 5 research reports were included in the previous study.
(2) In order to avoid bias, we will collect all relevant documents as comprehensively as possible.

As to the exploration of heterogeneity, post hoc subgroup analysis should be avoided as much as possible. Publishing this protocol will reduce potential biases associated with data mining, thereby helping to generate reliable evidence.

## Author contributions

**Conceptualization**: Hongjing Yang, Chuantao Zhang.

**Investigation**: Jianying Wu, Xiaohong Xie, Wei Xiao.

**Supervision**: Zhu Zeng, Keling Chen.

**Writing** – **original draft**: Wujun Wang, Xing An.

**Writing** – **review & editing**: Qingsong Huang, Wenjun Tang.
